# Pain‐related fear induces aberrant drop jump landing biomechanics in healthy and anterior cruciate ligament reconstructed females

**DOI:** 10.1002/ksa.12604

**Published:** 2025-02-04

**Authors:** Robert I. Dudley, Everett B. Lohman, Lida Gharibvand, Christopher S. Patterson

**Affiliations:** ^1^ School of Allied Health Professions Loma Linda University Loma Linda California USA; ^2^ Department of Physical Therapy Loma Linda University Loma Linda California USA; ^3^ Department of Physical Therapy Azusa Pacific University Azusa California USA

**Keywords:** ACL, biomechanics, drop jump, kinesiophobia, pain‐related fear

## Abstract

**Purpose:**

Rupture of the anterior cruciate ligament (ACL) is a prevalent and debilitating injury typically arising from aberrant biomechanics during landing or deceleration tasks. Pain‐related fear, a component of kinesiophobia, has been associated with poor functional outcomes and altered movement patterns in individuals with ACL reconstruction (ACLr), however, the influence of pain‐related fear on landing mechanics remains unclear. The purpose of this investigation was to examine the effects of pain‐related fear on landing movement patterns in a population of ACLr and healthy females.

**Methods:**

Thirty‐two females (15 recreationally active with a history of ACLr and 17 recreationally active with no history of ACLr) took part. Participants performed five trials of a drop jump (DJ) task (Baseline), underwent a pain stimulus (PS) familiarization task utilizing an electrical stimulus to induce pain‐related fear, and performed a subsequent round of DJs while under threat of PS (PS‐threat). Lower extremity and trunk kinematics, ground reaction force (GRF) data and muscle activation were analyzed.

**Results:**

At baseline, ACLr participants scored higher (21 ± 5.5) on the TSK‐11 compared to healthy participants (17 ± 3.4) (*p* = 0.007). For both groups, the PS intervention significantly increased pain‐related fear (ACLr *p* < 0.001; Healthy *p* < 0.001). When comparing baseline to PS‐threat trials, ACLr participants experienced a significant increase in peak GRF (*p* = 0.005), decreases in hip (*p* = 0.003) and knee (*p* = 0.005) flexion, decreased contact time (*p* = 0.006) and decreased muscle preactivation for all muscles tested (*p* < 0.05). Healthy participants experienced significant increases in peak GRF (*p* = 0.014) and decreased hip (*p* = 0.005) and trunk peak (*p* = 0.004) flexion.

**Conclusions:**

Pain‐related fear alters landing biomechanics in healthy and ACLr females. This may implicate pain‐related fear as a contributor to movement alterations commonly associated with ACL injury risk.

**Level of Evidence:**

Level III.

**Abbreviations**
ACL: anterior cruciate ligament
ACLr: anterior cruciate ligament reconstruction
BF: biceps femoris
DJ: drop jump
EMG: electromyography
e‐stim: electrical stimulation
GMax: gluteus maximus
GMed: gluteus medius
GRF: ground reaction force
PS: pain stimulus
RF: rectus femoris
RTP: return to play
TSK‐11: Tampa Scale of Kinesiophobia

## INTRODUCTION

Primarily arising from non‐contact movements such as landing and sudden decelerations [1, 5, 9, 16, 17, 25], ruptures of the anterior cruciate ligament (ACL) are both prevalent and debilitating. They account for over 50% of knee injuries and up to 4.24 billion dollars in annual economic impacts [3, 11, 12] with females between two and eight times more likely to be injured [3, 5, 11, 12]. Many injured individuals will go on to experience a subsequent ACL injury, and few return to prior levels of competition or physical activity [40]. In longitudinal research investigating the barriers to return to play (RTP) in the ACL reconstructed (ACLr) population, one of the most frequently cited reasons for failing to RTP was kinesiophobia [2, 14].

Kinesiophobia has also been associated with negative functional and biomechanical outcomes, particularly in those experiencing patellofemoral joint pain, low back pain, and ACLr [6, 19, 22, 32, 33, 35]. In the ACLr population, increases in kinesiophobia are associated with decreased peak knee, hip and trunk flexion during the execution of a drop jump (DJ) task [6, 35]. In terms of muscle activation, there is conflicting evidence regarding the influence of kinesiophobia. Recent investigations have demonstrated that kinesiophobia is associated with increased gluteus maximus (GMax) activation [35] and decreased vastus medialis activity [21] measured via surface electromyography (EMG) during the preactivation phase of landing tasks (defined in previous literature as either 50 ms [35] or 100 ms [21] prior to landing).

The DJ task is often used to assess ACL injury risk in both healthy (as a prospective screening mechanism) and ACLr populations (to assess RTP status and re‐injury risk) [24, 36]. An upright landing posture, observed in ACLr participants with high levels of kinesiophobia, resembles the well‐defined mechanism of injury for an ACL tear [9, 10]. A landing posture in which the ground reaction force (GRF) is not effectively distributed over larger joint ranges of motion increases shear and compressive forces at the tibiofemoral joint, increasing stress on the ligament and increasing the risk of a rupture [36]. Associations between kinesiophobia and biomechanics are supported in the literature [6, 19, 22, 32, 35]. Unfortunately, the aforementioned studies are correlational in design and prevent conclusions as to the direct effect of kinesiophobia on movement behaviour.

Kinesiophobia can arise from an injury or pain experience via increased sympathetic arousal [37, 41]. One component of kinesiophobia is pain‐related fear. The formation of pain‐related fear is reliant on the perception that the pain is threatening. Pain is interruptive in nature, demanding attention from the individual [39] and exposure to pain can, via the fear‐avoidance model [15, 38], lead to learned avoidant behaviours that occur not only during the pain experience, but when under threat of pain [39]. There is limited evidence of the deleterious effects of pain‐related fear on movement during fine motor tasks in healthy participants [13], however, to the best of our knowledge, there are no investigations examining the direct effects of pain‐related fear on gross motor behaviour in either healthy or clinical populations.

The purpose of this investigation is to examine the direct effects of acute pain‐related fear on landing movement patterns in a population of both ACLr and non‐injured, healthy females. Given the previous associations with aberrant landing patterns, we hypothesize that the introduction of pain‐related fear will alter landing movement patterns during a DJ task. Specifically, we hypothesize that pain‐related fear will lead to an upright landing posture consisting of reduced knee, hip and trunk flexion, along with increases to peak GRF, and decreases in muscle activation of the lower extremity during the preactivation phase of a drop jump in a cohort of ACLr female participants and a cohort of non‐injured females. Given the ACLr population's previous injury history and potential for increased baseline kinesiophobia, we also hypothesize that the magnitude of changes experienced by the ACLr group will be greater than the healthy group.

## METHODS

### Participants

Thirty‐two (15 ACLr and 17 Healthy) recreationally active females were recruited for this investigation (Table 1). For the ACLr group, participants were included if they were between 18 and 27 years old, had a history of a non‐contact, unilateral ACLr within the past 4 years, were cleared to return to full activity by a licensed healthcare professional, and were free of any lower extremity pain or injury within the previous three months. For the Healthy group, participants recruited were between 18 and 27 years old with no history of ACLr and were free of any lower extremity pain or injury within the previous three months [6, 35]. Prior to any testing, participants read and signed an Informed Consent Document approved by the University's Institutional Review Board.

**Table 1:** Participant demographics (mean ± SD).

	ACLr (*n* = 15)	Healthy (*n* = 17)	*p*
Age (years)	22.7 ± 2.6	22.5 ± 1.2	n.s.
Height (m)	1.65 ± 0.05	1.67 ± 0.07	n.s.
Mass (kg)	65.28 ± 10.36	60.91 ± 6.08	n.s.
TSK‐11	21 ± 5.5	17 ± 3.4	0.007*

Abbreviations: ACLr, anterior cruciate ligament reconstruction; SD, standard deviation; TSK‐11, Tampa Scale of Kinesiophobia.

* *p* < 0.05.

### Experimental procedures

Participants self‐reported demographic information regarding ACL injury and ACL surgical history (if applicable), physical activity levels and kinesiophobia. Physical activity was measured via the International Physical Activity Questionnaire, while kinesiophobia was measured via the Tampa Scale of Kinesiophobia (TSK‐11), an 11‐item survey with good internal consistency (*α* = 0.79) and test–retest reliability (intraclass correlation coefficient = 0.81, standard error of measurement = 2.54) [7, 20, 34, 41].

Subsequently, retroreflective markers were placed on the lower extremity and trunk of each participant. Individual markers were placed bilaterally on the first and fifth metatarsal heads, calcanei, medial and lateral malleoli, medial and lateral femoral epicondyles, anterior superior iliac spines, posterior superior iliac spines and acromion processes. Additional individual markers were placed on the sacrum, T8 spinous process, C7 spinous process, sternum, and xiphoid process. Rigid body marker clusters were affixed bilaterally to the foot, shank and thigh. An additional cluster was placed on the trunk at T3. Surface EMG electrodes were placed bilaterally on the GMax, gluteus medius (GMed), biceps femoris (BF), and rectus femoris (RF) according to established standards [8]. Prior to electrode placement, skin was shaved, abraded and cleaned with alcohol to reduce electrical impedance.

Kinematic data were recorded via an 18‐camera motion capture system (Qualisys) with a sampling frequency of 100 Hz, while two inground force platforms (AMTI) were used to record GRF data at 1000 Hz. EMG data were recorded using a wireless EMG system (Delsys) sampling at 2000 Hz.

Following a standardized dynamic warm‐up, including body weight squats, high knees, walking hamstring stretches and walking lunges, participants performed the baseline DJ task. Participants stood atop a 53‐cm box and were instructed to jump off the box and land with both feet simultaneously, and immediately jump vertically as high as possible [36]. Participants kept both hands on their hips throughout the task. Trials were excluded if the participant used their arms, lost balance, or did not land with both feet simultaneously. Participants performed the DJ with a 60 s rest between trials until five successful trials were recorded, with the middle three trials being used for analysis.

Upon completion of baseline testing, two silver/silver chloride (Ag/AgCl) electrodes were affixed to each participant's left hand. An electrical stimulation (e‐stim) unit (Chattanooga Continuum) was secured to the participant's left arm using an exercise device holder, connected to the electrodes, and controlled via a trigger by a researcher to deliver an electrocutaneous pain stimulus (PS). After placement, a participant‐specific calibration procedure was used to individually calibrate the level of PS. During the calibration, the e‐stim unit was set to 0 V and gradually increased until the participants reported a subjective pain rating of 8 on an 11‐point Likert scale ranging from ‘*no pain*’ to ‘*worst pain imaginable*’ [13]. The hand was chosen as the site of PS to not directly affect the musculature of the lower extremity with electrical stimulation, creating the threat of pain while not directly stimulating the muscles being examined.

**Figure 1:**
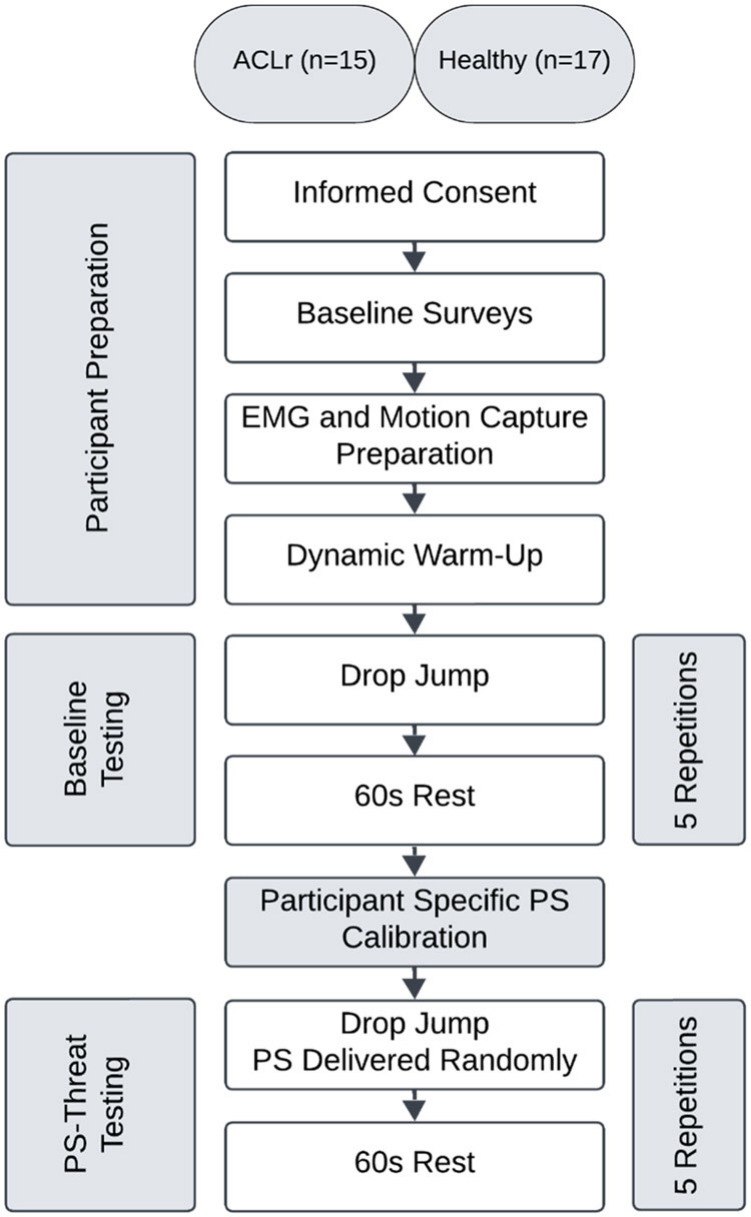
Flow chart depicting test setup and procedures. ACLr, anterior cruciate ligament reconstruction; EMG, electromyography; PS, pain stimulus.

Participants were instructed that their calibrated PS would be delivered during the landing phase of a random selection of DJ trials during the subsequent round of testing (i.e., some trials would be PS free, some trials a PS would be delivered). Additionally, a monitor placed in front of the participants displayed a ‘SHOCKS POSSIBLE’ message throughout the second round of testing. During the execution of the five DJ trials, a PS was delivered on two random trials. Trials in which no PS was delivered (PS‐threat) were utilized for analysis (Figure 1).

During both the baseline and PS‐threat trials, pain‐related fear was prospectively assessed by asking participants to rate their level of fear on an 11‐point Likert scale ranging from ‘*no fear*’ to ‘*extreme fear*’ immediately prior to the execution of each DJ trial.

### Data reduction

All data were analyzed using Visual 3D software (C‐Motion). For all variables, the participant's ACLr limb (ACLr group) or dominant limb (Healthy group) was used for analysis. Kinematic data were filtered using a fourth‐order lowpass Butterworth filter with a cutoff frequency of 12 Hz. Marker three‐dimensional positional data were used to calculate joint angles in the sagittal, frontal and transverse planes, with the pelvis modelled using the CODA method and hip joint centres defined using the Bell method [4, 18]. Joint angles were modelled as the motion of the distal segment relative to the proximal, apart from the trunk which was modelled relative to the lab. Peak joint angles were extracted during the landing phase of each task, defined as the period between initial contact and maximum knee flexion. Force plate data were filtered using a fourth‐order lowpass Butterworth filter with a cutoff frequency of 50 Hz. Vertical GRF was normalized to body weight (xBW). EMG signals were bandpass filtered at 20–350 Hz, smoothed using a 50 ms root mean square function [35], and normalized to the peak activity of each muscle during the execution of a maximum vertical jump normalization trial and expressed as a percentage (% MVC). Peak EMG data were extracted during two phases: the preactivation phase, defined as 50 ms prior to initial contact of landing on the force platforms [35] and during the loading phase, defined as the period between initial contact and maximum knee flexion. Initial contact was defined as the point at which vertical GRF exceeded 10 N [35].

### Statistical analysis

The mean ± standard deviation was computed for all quantitative variables. All data were tested for normality using the Shapiro–Wilk test and outliers were screened using boxplots. There were no outliers identified, and all cases were used for analysis. Mann–Whitney *U* tests were used to compare demographic variables at baseline due to non‐normality. Paired *t* tests and Wilcoxon signed‐rank tests were used where appropriate to compare baseline and PS‐threat trials in both the ACLr and Healthy populations. Independent *t* tests and Mann–Whitney *U* tests were used to analyze group differences. Cohen's *d* was calculated and classified as small (*d* = 0.2), medium (*d* = 0.5) and large (*d* ≥ 0.8), while *r* was calculated for non‐parametric tests and classified as small (*r* = 0.1), medium (*r* = 0.3) and large (*r* ≥ 0.5) [31]. A power calculation was performed using G*Power (Version 3.1.9.2; Heinrich‐Heine Universität) with a sample size of *n* = 32 required to provide 80% power at the 5% level of significance for an effect size of 0.25. All analyses were conducted using IBM SPSS Statistics 29 (IBM Corp.) with the level of significance set to 0.05.

## RESULTS

At baseline, ACLr participants (time post‐ACL tear 3.4 ± 0.7 years) scored higher on the TSK‐11 compared to healthy participants (p = 0.007) (Table 1). For both groups, the PS intervention significantly increased pain‐related fear (ACLr *p* < 0.001; Healthy *p* < 0.001). When comparing baseline to PS‐threat trials, ACLr participants experienced a significant increase in peak GRF (*p* = 0.005) and decreases in peak hip (*p* = 0.003) and knee (*p* = 0.005) flexion along with decreased contact time (*p* = 0.006) (Table 2). ACLr participants also saw significant decreases in muscle preactivation for the BF (*p* = 0.041), GMax (*p* = 0.009), GMed (*p* = 0.012) and RF (*p* = 0.005) and increases in GMed activation during the loading phase (*p* = 0.004) (Table 3). Healthy participants experienced a significant increase in peak GRF (p = 0.014) and a decrease in peak hip (*p* = 0.005) and trunk (*p* = 0.004) flexion (Table 2) but experienced no differences in muscle activation between conditions (Table 3).

**Table 2:** Pain‐related fear, GRF and kinematics (mean ± SD) during the landing phase of a DJ task.

Variable	ACLr (*n* = 15)				Healthy (*n* = 17)				Group comparison
	Baseline	PS‐threat	*p*	*d* a	Baseline	PS‐threat	*p*	*d* a	*p*
			Mean difference (CI)				Mean difference (CI)		
Pain‐related fear	1.1 ± 1.1	3.4 ± 1.7	<0.001* 2.3 (1.5–3.1)	0.79	0.2 ± 0.3	3.0 ± 1.9	<0.001* 2.7 (1.7–3.7)	1.42	n.s.
Peak GRF (xBW)	2.13 ± 0.34	2.37 ± 0.42	0.005* 0.24 (0.08–0.39)	0.86	2.58 ± 0.58	2.80 ± 0.54	0.014* 0.22 (0.05–0.39)	0.67	n.s.
Contact time (s)	0.60 ± 0.07	0.56 ± 0.06	0.006* −0.04 (−0.07 to −0.01)	0.83	0.56 ± 0.12	0.54 ± 0.12	n.s. −0.01 (−0.05 to 0.02)	0.23	n.s.
Hip flexion (°)	127.97 ± 21.34	118.15 ± 22.55	0.003* −9.82 (−15.61 to −4.02)	0.94	118.85 ± 20.35	111.93 ± 18.96	0.005* −6.91 (−11.42 to −2.40)	0.79	n.s.
Hip adduction (°)	−8.24 ± 3.75	−7.47 ± 4.25	n.s. 0.78 (−0.41 to 1.96)	0.36	−5.70 ± 6.04	−5.84 ± 5.11	n.s. −0.14 (−1.40 to 1.11)	0.06	n.s.
Knee flexion (°)	101.02 ± 12.22	96.17 ± 11.99	0.005* −4.85 (−7.93 to −1.77)	0.87	99.08 ± 10.37	99.61 ± 9.72	n.s. −2.46 (−5.09 to 0.17)	0.48	n.s.
Knee abduction (°)	−3.60 ± 4.94	−3.84 ± 5.25	n.s. −0.24 (−0.95 to 0.48)	0.18	−3.25 ± 5.08	−3.34 ± 5.65	n.s. −0.09 (−0.99 to 0.81)	0.05	n.s.
Trunk flexion (°)	42.92 ± 6.58	40.49 ± 5.39	n.s. −2.42 (0.26 to −5.11)	0.50	40.37 ± 11.78	36.10 ± 11.84	0.004* −4.27 (−6.99 to −1.55)	0.81	n.s.
Trunk lateral flexion (°)	1.96 ± 1.03	0.40 ± 3.17	n.s. −1.57 (0.21 to −3.33)	0.49	1.34 ± 2.15	0.82 ± 2.71	n.s. −0.52 (−1.65 to 0.60)	0.24	n.s.

Abbreviations: ACLr, anterior cruciate ligament reconstruction; CI, confidence interval; DJ, drop jump; GRF, ground reaction force; xBW, normalized to body weight.

* *p* < 0.05.

^a^ Cohen's *d*.

**Table 3:** EMG (mean ± SD) during the preactivation and landing phase of a DJ task.

Variable	ACLr (*n* = 15)				Healthy (*n* = 17)				Group comparison
	Baseline	PS‐threat	*p*	*r* a	Baseline	PS‐threat	*p*	*r* a	*p*
			Mean difference (CI)				Mean difference (CI)		
BF preactivation (% MVC)	21.09 ± 20.70	13.75 ± 12.56	0.041* −7.34 (−13.89 to −0.81)	0.06	17.39 ± 7.92	17.34 ± 8.05	n.s. −0.05 (−2.98 to 2.88)	0.53	n.s.
GMax preactivation (% MVC)	13.40 ± 7.42	10.38 ± 5.19	0.009* −3.02 (−5.40 to −0.65)	0.34	11.95 ± 5.79	13.11 ± 6.87	n.s. 1.16 (−0.45 to 2.77)	0.67	0.003*
GMed preactivation (% MVC)	21.13 ± 5.42	17.59 ± 6.39	0.012* −3.54 (−6.03 to −1.07)	0.06	19.56 ± 7.89	19.54 ± 6.09	n.s. −0.02 (−2.18 to 2.12)	0.65	0.043*
RF preactivation (% MVC)	33.85 ± 13.25	25.65 ± 10.26	0.005* −8.20 (−13.12 to −3.28)	0.33	37.09 ± 16.78	33.73 ± 14.73	n.s. −3.36 (−7.74 to 1.01)	0.72	n.s.
BF loading (% MVC)	19.66 ± 13.07	21.39 ± 17.10	n.s. 1.73 (−2.36 to 5.81)	0.02	28.53 ± 11.95	28.61 ± 11.70	n.s. 0.08 (−3.66 to 3.84)	0.03	n.s.
GMax loading (% MVC)	22.92 ± 21.39	24.03 ± 10.64	n.s. 1.11 (−6.22 to 8.43)	0.32	27.97 ± 11.13	31.59 ± 14.48	n.s. 3.62 (−1.30 to 8.55)	0.44	n.s.
GMed loading (% MVC)	21.86 ± 15.17	29.78 ± 22.96	0.004* 7.92 (1.76–14.09)	0.09	25.47 ± 14.21	25.09 ± 7.81	n.s. −0.38 (−5.95 to 5.21)	0.75	n.s.
RF loading (% MVC)	57.14 ± 22.12	55.01 ± 36.29	n.s. −2.13 (−14.19 to 9.92)	0.14	52.81 ± 13.02	52.61 ± 16.31	n.s. −0.2 (−5.80 to 5.41)	0.22	n.s.

Abbreviations: % MVC, percentage of the maximum voluntary contraction; ACLr, anterior cruciate ligament reconstruction; BF, biceps femoris; CI, confidence interval; DJ, drop jump; EMG, electromyography; GMax, gluteus maximus; GMed, gluteus medius; RF, rectus femoris; SD, standard deviation.

* *p* < 0.05.

^a^ Effect size (*r*).

## DISCUSSION

In support of our hypothesis, ACLr females demonstrated lower peak knee and hip flexion, higher GRF and lower contact time during landing. Additionally, ACLr participants demonstrated decreased muscle pre‐activation in all muscles tested. Healthy individuals also demonstrated lower peak hip and trunk flexion, and increased peak GRF, but changes in muscle activation were not observed.

The results of the current study suggest that pain‐related fear alters landing mechanics in such a way that may contribute to ACL stress during jump landing [29]. A decrease in trunk flexion during landing has been associated with greater use of the quads and increased tensile and shearing force on the ACL [36]. Lower peak knee flexion and hip flexion, combined with higher GRF, suggest a stiffer landing strategy, which has been associated with increased tibiofemoral shear and compressive forces that may result in an increased risk of ACL rupture [30]. When fearful, ACLr patients may adopt a landing strategy that increases the risk of injury.

That ACLr participants demonstrate decreased peak knee flexion and hip flexion is consistent with previous reports of alterations in movement patterns in the presence of fear. In a recent examination of ACLr females, Trigsted et al. demonstrated that increased self‐reported kinesiophobia was associated with decreased knee, hip, and trunk flexion during jump landing tasks [35]. Dudley et al. similarly found increased kinesiophobia to be associated with decreased knee and trunk flexion [6]. The results of the current study suggest a more direct influence of fear on landing mechanics. When ACLr participants completed a jump landing in the presence of a fearful stimuli, similar movement mechanics (decreased hip and knee flexion) were observed.

In terms of injury risk, the upright posture exhibited by both groups can significantly increase tibiofemoral shear and compressive forces [36], and decrease shock attenuation capacity [29], leading to increased risk of ACL rupture. The findings of the present study not only demonstrate relevance for ACL re‐injury rates but may help explain the increased risk of early onset Osteoarthritis in ACLr patients [27]. ACLr participants experienced a decrease in hip flexion of 9.8° (7.61% decrease), while healthy participants saw a decrease of 6.9° (5.81% decrease). The magnitude of these changes is greater than those reported by Tsai et al. when comparing pre‐ to post‐training kinematics during a DJ task [36]. These kinematic changes, coupled with the 11.27% (ACLr) and 8.53% (healthy) increase in peak GRF are indicative of movement patterns associated with ACLr injury risk [28, 29].

It is possible that participants restricted joint motion to protect the body from pain (as induced via PS); however, in doing so, they engaged in potentially injurious movements. When an individual experiences pain, they may associate negative emotions with the experience. If the individual perceives the pain as threatening, they may engage in avoidant behaviours to mitigate the pain. These avoidant behaviours can persist long after the initial pain has subsided, potentially leading to the recurrence of the pain experience [38].

During the preactivation phase of landing, ACLr participants experienced significant decreases in preparatory muscle activation across all muscles tested when comparing baseline to PS‐threat conditions. These findings are supported by those of Ohji et al., who demonstrated that kinesiophobia was associated with a decrease in vastus medialis preparatory activity during a single‐leg landing task [21]. Our findings contrast those of Trigsted et al., in which kinesiophobia was associated with increases in GMax preparatory activity [35]. Preparatory muscle activity functions to effectively prepare the muscles and associated tendons for the large increases in necessary muscle contractions post‐landing [26]. Decreases in preparatory muscle activation may diminish the ability of the participant to distribute the landing forces and control the limb segments as desired. Interestingly, healthy participants did not experience any significant changes to preparatory muscle activity, indicating that baseline kinesiophobia and the presence of previous injury may be exacerbating factors influencing muscle activity.

We demonstrated jump landing mechanics changes in both healthy and ACLr females. These findings support those of Karos et al., who established that pain‐related fear affects joystick manoeuvres in pain‐free individuals [13]. After acquiring pain‐related fear via an electrical stimulus during initial rotation tasks, participants performed subsequent rotations more forcefully, more accurately, and faster. While task accuracy was not measured in the present investigation, participants in the ACLr group did have significantly shorter contact times when comparing baseline to PS‐threat trials. As Karos et al., suggested, the faster execution of movement may be a strategy employed to hasten the completion of the task and potential PS [13]. During the execution of a DJ task, this decrease in contact time may eliminate the ability for participants to fully utilize the sagittal plane range of motion and potentially engage in protective movement strategies which encourage increasing trunk, knee, and hip flexion. The fear‐avoidance model provides further evidence regarding the alterations in movement experienced in both groups [15]. In the ACLr population, pain‐related fear has been shown to be present throughout the post‐injury and rehabilitation period and is associated with injurious movement patterns [14, 23]. The present investigation adds to the body of literature by incorporating experimental PS and movement analysis in addition to baseline TSK‐11 measurements. The results of the current investigation also suggest that clinicians and practitioners involved with ACL injury prevention and ACLr rehabilitation protocols should consider including techniques to test for and reduce kinesiophobia and pain‐related fear given their effects on potentially injurious movement patterns.

There are limitations to consider when interpreting our findings. The sample of participants was limited to recreationally active females between 18 and 27 years of age. These findings may not be generalizable beyond this population, and it is unclear if males would respond differently. Furthermore, we did not investigate the lasting effects of the pain‐related fear. It is possible that subsequent executions of the DJ task would revert to less injurious movement patterns upon removal of the threat of PS. Additionally, our experimental PS only mimics real‐world pain scenarios; pain, fear response, and coping mechanisms may be different outside the laboratory environment. Finally, we only investigated a dual‐limb task, and future investigations should consider examining single‐limb tasks to fully explore ACL risk.

## CONCLUSION

Pain‐related fear significantly alters lower extremity and trunk kinematics and GRF in both healthy and ACLr females and significantly alters preparatory muscle activity in ACLr females. These findings indicate that pain‐related fear is a powerful factor which may exacerbate movement alterations associated with ACL injury risk. Future investigations should examine the direct effects of pain‐related fear on additional unilateral tasks associated with ACL injury.

## AUTHOR CONTRIBUTIONS

All authors contributed to the study conception and design. Material preparation, data collection and analysis were performed by R. Dudley, E. Lohman and C. Patterson. Statistical analysis was performed by R. Dudley and L. Gharibvand. The first draft of the manuscript was written by R. Dudley and all authors commented on previous versions of the manuscript. All authors read and approved the final manuscript.

## CONFLICT OF INTEREST STATEMENT

The authors declare no conflicts of interest.

## ETHICS STATEMENT

All research activities were approved by the University Institutional Review Board. All participants provided written informed consent prior to participation.

## Data Availability

The data that support the findings of this study are available from the corresponding author upon reasonable request.

## References

[1] Acevedo RJ , Rivera‐Vega A , Miranda G , Micheo W . Anterior cruciate ligament injury: Identification of risk factors and prevention strategies. Curr Sports Med Rep. 2014;13(3):186–191. 24819011 10.1249/JSR.0000000000000053

[2] Ardern CL , Webster KE , Taylor NF , Feller JA . Return to sport following anterior cruciate ligament reconstruction surgery: a systematic review and meta‐analysis of the state of play. Br J Sports Med. 2011;45(7):596–606. 21398310 10.1136/bjsm.2010.076364

[3] Arendt E , Dick R . Knee injury patterns among men and women in collegiate basketball and soccer. NCAA data and review of literature. Am J Sports Med. 1995;23(6):694–701. 8600737 10.1177/036354659502300611

[4] Bell AL , Pedersen DR , Brand RA . A comparison of the accuracy of several hip center location prediction methods. J Biomech. 1990;23(6):617–621. 2341423 10.1016/0021-9290(90)90054-7

[5] Boden BP , Dean GS , Feagin JA , Garrett WE . Mechanisms of anterior cruciate ligament injury. Orthopedics. 2000;23(6):573–578. 10875418 10.3928/0147-7447-20000601-15

[6] Dudley RI , Lohman EB , Patterson CS , Knox KG , Gharibvand L . The relationship between kinesiophobia and biomechanics in anterior cruciate ligament reconstructed females. Phys Ther Sport. 2022;56:32–37. 35717878 10.1016/j.ptsp.2022.06.002

[7] George SZ , Lentz TA , Zeppieri G , Lee D , Chmielewski TL . Analysis of shortened versions of the Tampa Scale for Kinesiophobia and pain catastrophizing scale for patients after anterior cruciate ligament reconstruction. Clin J Pain. 2012;28(1):73–80. 21677565 10.1097/AJP.0b013e31822363f4 PMC3703641

[8] Hermens HJ , Freriks B , Disselhorst‐Klug C , Rau G . Development of recommendations for SEMG sensors and sensor placement procedures. J Electromyography Kinesiol. 2000;10(5):361–374. 10.1016/s1050-6411(00)00027-4 11018445

[9] Hewett TE , Myer GD . The mechanistic connection between the trunk, hip, knee, and anterior cruciate ligament injury. Exerc Spotr Sci Rev. 2011;39(4):161–166. 10.1097/JES.0b013e3182297439 PMC4168968 21799427

[10] Hewett TE , Myer GD , Ford KR , Paterno MV , Quatman CE . Mechanisms, prediction, and prevention of ACL injuries: cut risk with three sharpened and validated tools. J Orthop Res. 2016;34(11):1843–1855. 27612195 10.1002/jor.23414 PMC5505503

[11] Kaeding CC , Léger‐St‐Jean B , Magnussen RA . Epidemiology and diagnosis of anterior cruciate ligament injuries. Clin Sports Med. 2017;36(1):1–8. 27871652 10.1016/j.csm.2016.08.001

[12] Kaeding CC , Pedroza AD , Reinke EK , Huston LJ , Spindler KP , Amendola A , et al. Risk factors and predictors of subsequent ACL injury in either knee after ACL reconstruction. Am J Sports Med. 2015;43(7):1583–1590. 25899429 10.1177/0363546515578836 PMC4601557

[13] Karos K , Meulders A , Gatzounis R , Seelen HAM , Geers RPG , Vlaeyen JWS . Fear of pain changes movement: motor behaviour following the acquisition of pain‐related fear. Eur J Pain. 2017;21(8):1432–1442. 28444803 10.1002/ejp.1044

[14] Kvist J , Ek A , Sporrstedt K , Good L . Fear of re‐injury: a hindrance for returning to sports after anterior cruciate ligament reconstruction. Knee Surg Sports Traumatol Arthrosc. 2005;13(5):393–397. 15703963 10.1007/s00167-004-0591-8

[15] Lethem J , Slade PD , Troup JDG , Bentley G . Outline of a Fear‐Avoidance Model of exaggerated pain perception—I. Behav Res Ther. 1983;21(4):401–408. 6626110 10.1016/0005-7967(83)90009-8

[16] Li G , DeFrate LE , Sun H , Gill TJ . In vivo elongation of the anterior cruciate ligament and posterior cruciate ligament during knee flexion. Am J Sports Med. 2004;32(6):1415–1420. 15310565 10.1177/0363546503262175

[17] Micheo W , Hernández L , Seda C . Evaluation, management, rehabilitation, and prevention of anterior cruciate ligament injury: current concepts. PM R. 2010;2(10):935–944. 20970763 10.1016/j.pmrj.2010.06.014

[18] Moore KD , Hawke AL , Carey RE , Wu JZ , Breloff SP . Agreement of hip kinematics between two tracking marker configurations used with the coda pelvis during ergonomic roofing tasks. J Mech Med Biol. 2023;23(3). 10.1142/s021951942350015x PMC10285509 37361026

[19] Nedder VJ , Raju AG , Moyal AJ , Calcei JG , Voos JE . Impact of psychological factors on rehabilitation after anterior cruciate ligament reconstruction: a systematic review. Sports Health. 2024. Epub 2024 Jul 23. 10.1177/19417381241256930 PMC11569572 39041333

[20] Nwachukwu BU , Adjei J , Rauck RC , Chahla J , Okoroha KR , Verma NN , et al. How much do psychological factors affect lack of return to play after anterior cruciate ligament reconstruction? A systematic review. Orthop J Sports Med. 2019;7(5):2325967119845313. 31205965 10.1177/2325967119845313 PMC6537068

[21] Ohji S , Aizawa J , Hirohata K , Ohmi T , Mitomo S , Koga H , et al. Association between landing biomechanics, knee pain, and kinesiophobia in athletes following anterior cruciate ligament reconstruction: a cross‐sectional study. PM R. 2023;15(5):552–562. 35474441 10.1002/pmrj.12827

[22] de Oliveira Silva D , Barton CJ , Briani RV , Taborda B , Ferreira AS , Pazzinatto MF , et al. Kinesiophobia, but not strength is associated with altered movement in women with patellofemoral pain. Gait Posture. 2019;68:1–5. 30408709 10.1016/j.gaitpost.2018.10.033

[23] Paterno MV , Flynn K , Thomas S , Schmitt LC . Self‐reported fear predicts functional performance and second ACL injury after ACL reconstruction and return to sport: a pilot study. Sports Health. 2018;10(3):228–233. 29272209 10.1177/1941738117745806 PMC5958451

[24] Pollard CD , Sigward SM , Powers CM . ACL injury prevention training results in modification of hip and knee mechanics during a drop‐landing task. Orthop J Sports Med. 2017;5(9):2325967117726267. 28959697 10.1177/2325967117726267 PMC5593213

[25] Quatman CE , Kiapour AM , Demetropoulos CK , Kiapour A , Wordeman SC , Levine JW , et al. Preferential loading of the ACL compared with the MCL during landing: a novel in sim approach yields the multiplanar mechanism of dynamic Valgus during acl injuries. Am J Sports Med. 2014;42(1):177–186. 24124198 10.1177/0363546513506558 PMC3927458

[26] Santello M . Review of motor control mechanisms underlying impact absorption from falls. Gait Posture. 2005;21(1):85–94. 15536038 10.1016/j.gaitpost.2004.01.005

[27] Sepúlveda F , Sánchez L , Amy E , Micheo W . Anterior cruciate ligament injury: return to play, function and long‐term considerations. Curr Sports Med Rep. 2017;16(3):172–178. 28498226 10.1249/JSR.0000000000000356

[28] Sheehan FT , Sipprell WH , Boden BP . Dynamic sagittal plane trunk control during anterior cruciate ligament injury. Am J Sports Med. 2012;40(5):1068–1074. 22383659 10.1177/0363546512437850 PMC3582351

[29] Shimokochi Y , Ambegaonkar JP , Meyer EG , Lee SY , Shultz SJ . Changing sagittal plane body position during single‐leg landings influences the risk of non‐contact anterior cruciate ligament injury. Knee Surg Sports Traumatol Arthrosc. 2013;21(4):888–897. 22543471 10.1007/s00167-012-2011-9 PMC12269904

[30] Shimokochi Y , Shultz SJ . Mechanisms of noncontact anterior cruciate ligament injury. J Athl Train. 2008;43(4):396–408. 18668173 10.4085/1062-6050-43.4.396 PMC2474820

[31] Sullivan GM , Feinn R . Using effect size—or why the P value is not enough. J Grad Med Educ. 2012;4(3):279–282. 23997866 10.4300/JGME-D-12-00156.1 PMC3444174

[32] Thomas JS , France CR . Pain‐related fear is associated with avoidance of spinal motion during recovery from low back pain. Spine. 2007;32(16):E460–E466. 17632385 10.1097/BRS.0b013e3180bc1f7b

[33] Thomas JS , France CR , Lavender SA , Johnson MR . Effects of fear of movement on spine velocity and acceleration after recovery from low back pain. Spine. 2008;33(5):564–570. 18317203 10.1097/BRS.0b013e3181657f1a

[34] Tkachuk GA , Harris CA . Psychometric properties of the Tampa Scale for Kinesiophobia‐11 (TSK‐11). J Pain. 2012;13(10):970–977. 23031396 10.1016/j.jpain.2012.07.001

[35] Trigsted SM , Cook DB , Pickett KA , Cadmus‐Bertram L , Dunn WR , Bell DR . Greater fear of reinjury is related to stiffened jump‐landing biomechanics and muscle activation in women after ACL reconstruction. Knee Surg Sports Traumatol Arthrosc. 2018;26(12):3682–3689. 29700560 10.1007/s00167-018-4950-2

[36] Tsai L‐C , Ko Y‐A , Hammond KE , Xerogeanes JW , Warren GL , Powers CM . Increasing hip and knee flexion during a drop‐jump task reduces tibiofemoral shear and compressive forces: implications for ACL injury prevention training. J Sports Sci. 2017;35(24):2405–2411. 28006992 10.1080/02640414.2016.1271138

[37] Vlaeyen JWS , Kole‐Snijders AMJ , Rotteveel AM , Ruesink R , Heuts PHTG . The role of fear of movement/(re)injury in pain disability. J Occup Rehabil. 1995;5(4):235–252. 24234727 10.1007/BF02109988

[38] Vlaeyen JWS , Linton SJ . Fear‐avoidance and its consequences in chronic musculoskeletal pain: a state of the art. Pain. 2000;85(3):317–332. 10781906 10.1016/S0304-3959(99)00242-0

[39] Vlaeyen JWS , Morley S , Crombez G . The experimental analysis of the interruptive, interfering, and identity‐distorting effects of chronic pain. Behav Res Ther. 2016;86:23–34. 27614948 10.1016/j.brat.2016.08.016

[40] Wiggins AJ , Grandhi RK , Schneider DK , Stanfield D , Webster KE , Myer GD . Risk of secondary injury in younger athletes after anterior cruciate ligament reconstruction. Am J Sports Med. 2016;44(7):1861–1876. 26772611 10.1177/0363546515621554 PMC5501245

[41] Woby SR , Roach NK , Urmston M , Watson PJ . Psychometric properties of the TSK‐11: a shortened version of the Tampa Scale for Kinesiophobia. Pain. 2005;117(1):137–144. 16055269 10.1016/j.pain.2005.05.029

